# Visual inspection with acetic acid and Lugol's iodine in cervical cancer screening at the general referral hospital Kayembe in Mbuji-Mayi, Democratic Republic of Congo

**DOI:** 10.11604/pamj.2016.23.64.8450

**Published:** 2016-03-01

**Authors:** Banza Kamba Desire, Cilundika Mulenga Philippe, Kabengele Thierry, Kitenge Wa Momat Félix, Gilbert Utshudienyema Wembodinga, Kakudji Luhete Prosper, Luboya Numbi Oscar

**Affiliations:** 1 Department of Public Health, Faculty of Medicine, University of Lubumbashi, DR Congo; 2 General Referal Hospital Kayembe, Mbuji-Mayi, DR Congo; 3 Department of Gynaecology and Obstetrics, Faculty of Medicine, University of Lubumbashi, DR Congo; 4 School of Public Health, University of Kinshasa, DR Congo

**Keywords:** Cervical cancer, screening, visual methods, VIA, VILI, Mbuji-Mayi, Democratic Republic of Congo

## Abstract

**Introduction:**

Cervical cancer is the leading cause of morbidity and mortality of women from cancer in the developing World. It is the primary cause of reduced life expectancy in Sub-Saharan countries such as Democratic Republic of Congo. The aim of this work was to determinate the socio-demographic profile of women with precancerous and cancerous lesions of the cervix, to determinate the frequency of VIA and VILI positive cases and to show the challenges that can be faced in managing patients with abnormalities in the city of Mbuji-Mayi in the Democratic Republic of Congo.

**Methods:**

As part of its activities, the “Association de Lutte contre le Cancer du Col utérin” (ALCC) organized a community outreach followed by free voluntary testing for cervical cancer for two weeks (26^th^March to 10^th^ April 2011) at the General Referral Hospital Kayembe in Mbuji-Mayi (Democratic Republic of Congo).

**Results:**

A total of 229 women were examined. 38% of tests (VIA + VILI) were positive with 6 clinically suspected cases of invasive cancer at stage 1 (7% of cases). Nearly 70% of patients were still of childbearing age and had started their first sexual intercourse before 18 years of age and 86% of cases were multiparous. Given the material, financial and technical constraints, 75% of patients were placed in a monitoring program of 9 months to 1 year (= expectation and another test) while 11% of them were selected for a biopsy to be locally practiced and sent to the pathologist. Nearly 8% of the cases were candidates for hysterectomy.

**Conclusion:**

Given the difficulties encountered and the frequency of positive tests, we recommend another study with a larger sample, improved working conditions (mainly equipment) and the association of another test such as the Human Papilloma Virus (HPV) test.

## Introduction

Cervical cancer is the leading cause of morbidity and mortality of women from cancer in the developing World. It is the primary cause of reduced life expectancy in Sub-Saharan countries such as Democratic Republic of Congo, South East Asia and Latin America [1]. However, it is a disease that can be prevented by early detection techniques that are Papanicolaou cytology (Pap smears), visual methods (visual inspection of the cervix after application of acetic acid “VIA” or iodine solution “VILI”) and recent techniques for detecting viral DNA or proteins E6, E7 in human papilloma virus recognized as the main causative agent of cervical cancer. However, despite all the technological advances that have significantly reduced the incidence of the disease in the northern countries, similar trends have not been observed in developing countries [2]. Thus, in many countries, since the 80s, efforts have been multiplied to find ways to combat it, but did not come in reducing mortality from cervical cancer. According to Kitchener [3], the failure of the implemented programs is due to lack of funds, lack of access to rural areas where the majority of the population in our country lives, lack of awareness and information for screening and monitoring difficulties. Given the disappointing results obtained with conventional cytology in poor settings because of various hindrances, alternative methods of cancer screening based on the direct examination of the cervix after application of 5% acetic acid (VIA) or iodine solution (VILI) appear promising in this context. These methods are very inexpensive, easy to apply and to learn even by intermediate paramedics and give immediate results, which allow faster treatment. VIA is comparable to the Pap smear in detecting cervical intraepithelial high- grade intra-epithelial lesions of the cervix [4]. This study, therefore, claims to be the beginning of a response to this major public health concern and a first experience in the prevention of cervical cancer in an environment with very limited resources like the Health District of Diulu in Mbuji-Mayi (Democratic Republic of Congo). The aim of this work was to determinate the socio-demographic profile of women with precancerous and cancerous lesions of the cervix, to determinate the frequency of VIA and VILI positive cases and to show the challenges faced by the team in managing the patients with cervical abnormalities in the city of Mbuji-Mayi in the Democratic Republic of Congo.

## Methods

The General Referral Hospital Kayembe serves as the hospital of reference for 33 health centres of the health district of Diulu in the city of Mbuji-Mayi that is located in the central part of the Democratic Republic of Congo. It is important to know that the city is 1,000 Km away from Kinshasa, the capital and from Lubumbashi, the second largest city of the country. There is no medical facility that provides pathological diagnosis in Mbuji Mayi. Therefore samples have to be sent to pathologists in these two cities. 317139 people live in that area according to the last estimates, 10% from which should be expected at the Hospital. The Department of Obstetrics and Gynaecology is partially functional. It is the most developed Department with approximately 100 deliveries per month. The gynaecological activities are less developed for technical reasons (only one part-time gynaecologist working only every Saturday and lack of adequate equipments). So, we had to train a young doctor and the two nurses who were full-time employed there. This was a cross-sectional clinical descriptive study in which the sample consisted of all voluntary women aged of at least 20 years, having started sexual activity at least one year before, not being pregnant or menstruating. These women were first briefed on the disease's theory in order to obtain informed consent for them to be examined and possibly treated. An awareness campaign on radio and television as well as through community outreach (health workers in the health district of Diulu) was organized to bring the target population to be screened for free at the hospital during our activities which lasted two weeks (26^th^ March to 10^th^ April 2011) because of financial constraints. Before embarking in the study, permission was sought and granted by the local health authority. Financial constraints such as travel money or screening and treatment costs were not an issue in this study as all the participants could get to hospital even on foot (maximum distance of 3 miles) and the screening and the treatment were freely provided. To sustain this activity at the hospital, a local team was trained including a doctor and two nurses. All the volunteer participants were asked to respond to questions related to their age, educational level, last menstrual period (LMP), marital status, parity and complaints related to cervical cancer. After screening, we noted the results of VIA and VILI and the proposed management of each case.

The following steps were followed:

*Informed consent*: before undertaking the screening and treatment procedures, we had to explain it, why we wanted to do it, what conclusion we were going to make from it and the course of action we were going to take accordingly. Each participant was free to accept or to refuse the examination.

*An interview*: this was a quantitative structured questionnaire that each participant had to answer to. The questions were about the age, the marital status, the level of education, the age at the first intercourse, the last menstrual period (LMP), the parity and the reason for consulting.

*A direct examination of the cervix;* an examination of the cervix after application of acetic acid (5% solution); an examination of the cervix after application of Lugol's iodine

*The announcement of the results and the proposed course of action and indication*: the result was immediately announced. Then the course of action taken was discussed as follow: total hysterectomy was performed in patients aged over 40 years with both VIA and VILI positive tests, with a large suspicious iodine- negative area and who no longer had a desire of pregnancy and clinically there was a suspect lesion of cervical cancer strictly localized at the cervix. If there was a desire for motherhood, a biopsic and therapeutic cold knife conization were recommended and performed. In the absence of a cryosurgical device or LEEP, patients with small lesions were asked to return between nine months and one year for a control test. Regarding women with a negative test, we recommended testing them every 5 years. For this descriptive study, only frequency, average and percentage were calculated.

## Results

In total we received 229 women at the screening site of the gynaecological department after a week of awareness campaigns through the radio, the television, churches and door-to-door. All of them accepted to be screened after a face-to-face explanation of all the procedures. The age ranged between 22 and 67 years. There were 88VIA and VILI positive tests (38% of the women) and 6 (7%) clinically suspected cases of invasive cancer, stage 1. The average age for first sexual intercourse was 17.5 ± 0.7years. As shown in Table 1 on socio demographic characteristics, nearly 70% of the cases were aged between 30 and 49. Seventy per-cents of them were single and were educated at the high school level. The majority of the cases (62% and not 50% because 18 cases failed to provide this information) started their first sexual intercourse before 18 years of age. 70% of the cases were still menstruating. All the multiparous cases represented 86%. The vast majority of cases (84%) had complaints not related to cervical cancer (Table 2). Table 3 presents the distribution of cases according to the undertaken action. Almost the three quarter of the cases were put on the follow up programme (9 to 12 months) whereas 12.5% beneficiated of a diagnostic biopsy. We performed a total hysterectomy on 8% of the cases aged over 40 with a suspected lesion that we clinically classified as invasive cervical cancer at the stage I. However, we were obliged to perform a cold knife conisation on 3 cases with large suspected lesions, but having a desire of maternity.

**Table 1:** Scio demographics characteristics of cases

Variable	Number of cases (n = 88)	Percent
**Age (years)**		
20-29	10	11.4
30-39	29	33.0
40-49	32	36.4
50-59	13	14.8
≥60	4	4.6
**Marital Status**		
Married	70	79.5
Divorced	7	8.0
Widowed	10	11.4
Other	1	1.1
**Level of Study**		
Did not go to school	3	3.4
Primary	17	19.3
High School	62	70.5
College/University	6	6.8
**Age at 1st Intercourse**		
Before 18 years of age	44	50.0
After 18 years of age	26	29.5
Failed	18	20.5
**Last Menstrual Period**		
Less than 12 months	62	70.5
More than 12 months	26	29.5
**Parity**		
0	5	6.0
1-2	7	8.0
3-4	22	25.0
≥5	54	61.0

**Table 2:** Distribution of cases according to the main complaints

Main complaints	Number of cases	Percent
Not related to cervical cancer	74	84.0
Related to cervical cancer	14	16.0
**Total**	88	100

**Table 3:** Distribution of cases according to the undertaken action

Action	Number of cases	Percent
Biopsy	11	12.5
Cold Knife Cone	3	3.4
Expectation and Control test after 9 months	24	27.3
Expectation and Control test after 12 months	43	48.8
Total hysterectomy	7	8.0
**Total**	88	100

## Discussion

In this study there were 38% positive tests. This figure is considerably higher than that reported by Lawrence [5] who had 13% positive tests using IVA alone. This could be due to the fact that they worked on a larger sample (954 women screened) while there were only 229 women in our study. The lack of experience of the local team is not to be overlooked as well. We also combined IVA and VILI which could have had an impact on our results. The average age of first intercourse was 17.5±0.7 years. In France, it is almost the same (17.6 years) according to the National Institute of Demographic studies [6]. According to the global survey on sexuality conducted in 52 countries in five continents [7], contrary to popular belief, the age of first intercourse remained stable between 15 and 19 years, with, however, large regional disparities. The age at first intercourse before 18 (70% of cases in our study) is a known risk factor for cancer of the cervix [8]. The multiparity (86% of cases in our study) is also a predisposing factor for the development of the disease [9]. We noted nearly 85% of positive tests between 30 and 59 years and it is from 30 years that the frequency of cases increased. Kumar and colleagues postulated that a woman can develop cervical dysplasia at any age, but usually between 25 and 35 years [10]. From our study, it is not possible to hypothesize at what age our patients developed cervical dysplasia. Regarding the level of education of the patients, the majority had high school education. Our sample being small, we did not have many women of higher or university level. Our framework did not provide enough comfort, hence, perhaps did not attract the latter category of highly educated women. Our findings are, however, useful for awareness campaigns as the majority of women who can read and write can more easily understand the information messages about the disease and its prevention and explain to others and get tested. Women with low educational level may have less knowledge and little control regarding the decisions on sexual and reproductive health matters [11].

Married women accounted for nearly 80% of cases. Polygamy is a common practice in the area of the DR Congo. Even women with one partner can easily get in contact with the HPV virus contracted by their partner during unprotected extramarital relationships. In corroboration a study in South Africa indicated that sexual activity is associated with an increased risk of cervical cancer [12]. According to the same study, singles have more opportunities to have multiple sexual partners and therefore more likely to contract the disease. It is not surprising that 84% of patients in our study had no complaints in relation to cervical cancer such as an history of bad smelly vaginal discharge, post coital bleeding. Indeed, screening is aimed at assumed healthy people. Regarding the management of cases, cryosurgery which could have been implemented for small lesions in our study, this technique “see and treat” has been noted to carry the risk of unnecessarily treating some women as noted by many authors [13–16]. The main challenge has been to discuss and perform an abdominal total hysterectomy without a pathological diagnosis. A biopsy would be ideal, but taking into account the high cost of a pathological examination in Kinshasa and Lubumbashi and putting together the socio-demographical profile of patients, we performed 8 hysterectomies without complications putting ourselves to critics. In the absence of pathological diagnosis and considering the slow progression of the lesions as described in the natural history of cervical cancer [16], with the exception of patients with immune deficiency syndrome (HIV positive), several studies have shown an increased risk of precancerous cervical lesions [16]. Nine to 12 months of observation was justified as an option in case of doubt or for cases that had not to undergo conisation immediately given the viral clearance is 70-80%. As you can see we did work in an environment with very scarce resources. In order to improve the current services, we need to ameliorate the comfort on the site in order to attract more participants that may have been reluctant mainly among intellectual women; more training and supervision are needed for the personal in the site; a good “see and treat” system using a cryosurgical system treatment needs to be added to avoid losing some participants who may forget to come back for the follow up 9 to 12 months later or may underestimate the condition because of the lack of symptoms in early stages of the disease. A colposcopic examination of the cervix eventually followed by biopsy and pathological diagnosis is ideal [17].

## Conclusion

Despite many limitations of our screening techniques, the high number of positive tests is greatly disturbing, as well as that of invasive cancer clinically suspected. The multi-parity remains a considerable risk factor as well as an early age at first intercourse. Most cases were found among women aged from 30 years. We suggest a further study with a larger sample to be undertaken, a more trained personal, a more comfortable working environment and more adequate equipment: cryosurgery, Leep and colposcopy device. It would be useful to associate the visual methods and a rapid HPV test.

### What is known about this topic

- The natural history of cervical cancer
- The methods of screening for it
- How to treat precancerous lesions

### What this study adds

- This is the first study in this topic in the region (Mbuji-Mayi/D.R.Congo)
- This study shows how is challenging to treat patients with the disease in settings with very limited resources
- The study constitutes a contribution to a global plea for a cervical cancer prevention programme in the Democratic Republic of Congo
